# Undernutrition among tribal children in Palghar district, Maharashtra, India

**DOI:** 10.1371/journal.pone.0212560

**Published:** 2019-02-27

**Authors:** Soumitra Ghosh, Sarika A. Varerkar

**Affiliations:** 1 Centre for Health Policy, Planning and Management, School of Health Systems Studies, Tata Institute of Social Sciences, Mumbai, India; 2 School of Health Systems Studies, Tata Institute of Social Sciences, Mumbai, India; BITS Pilani, INDIA

## Abstract

**Background:**

Maharashtra is the richest Indian state. However, prevalence of undernutrition is unacceptably high in Maharashtra, particularly among the tribal children. In 2005, child malnutrition claimed as many as 718 lives in one single district namely Palghar. Even after a decade of double-digit economic growth, in 2016, more than 600 children died due to under-nutrition in the same district. The state then announced a slew of measures to address child malnutrition in tribal dominated areas. There has not been any study to check whether the nutritional scenario has improved since then in Palghar. Hence, the present study was undertaken to assess the magnitude of under-nutrition among the tribal children under six years of age, their dietary pattern and food practices in the Vikramgad block of Palghar District.

**Methods:**

The study is based on a survey conducted among the 375 tribal households with children aged between 1 and 6 during April-June 2017. The sample was selected through a two-stage stratified random sampling. Both height and weight measurements were taken from each of the 375 children. The assessment of their nutritional status was carried out using the 2006 WHO Child Growth Standard. Besides, multivariate logistic regression models were employed to understand the independent effects of predictor variables on stunting, wasting and underweight.

**Results:**

Our study level estimates suggest that 59% of children were stunted. The overall prevalence of wasting and underweight was 20% and 53% respectively. The dietary recall data revealed 83% of the children had consumed food belonging to only 3 groups. Further, the most common food eaten by the children was rice and dal (pulses). Only 13% of the children achieved a minimum level of diet diversity.

## Introduction

Development is not synonymous with growth as development is about expanding the freedom and capabilities of the disadvantaged, thereby improving the overall quality of life [1]. Based on this understanding, Maharashtra, is a classic case of lack of development as reflected in its unacceptably high level of malnutrition among the children. The point to be noted is that Maharashtra is the richest Indian state with GDP of $430 billion, which is equivalent to the economy of Norway [2]. In fact, if Maharashtra were to declare independence, it would be classified as a middle income country and world’s twelfth most populous nation. While the state’s per capita income has doubled since 2004 as a result of sustained high economic growth, its nutritional status has not made commensurate progress during this period. Recent nutritional assessments show that 35% of the children under 5 were stunted, 18% were wasted and 25% were underweight in 2013–14 [3]. Another estimate provided by National Family Health Survey 2015–16 suggests that 34% of children under age five years were stunted, or too short for their age, indicating that they had been chronically undernourished, 26 percent were wasted which may be a result of recent inadequate food intake or a recent illness causing weight loss, and 9% were severely wasted and 36 percent were underweight, which takes into account both chronic and acute undernutrition [4].

It is disconcerting to note that, despite various nutrition programmes including the flagship Maharashtra Nutrition Mission (launched in 2005), undernutrition scenario in Maharashtra remains grim. A comparison of anthropometric indicators for children under five years old, using the 3^rd^ and 4^th^ rounds of National Family Health Survey (NFHS) survey 2015–2016 and 2005–06, reveals that though stunting has declined from 46.3% to 34.4%, wasting rates increased alarmingly from 16.5% to 25.6%–an increase of almost ten percentage points during the said period. Further, severe wasting has nearly doubled and underweight rate (36% and 37% in 2015–16 and 2005–06 respectively) has remained almost unchanged in the last 10 years. The global comparison of malnutrition shows that the prevalence of undernutrition in Maharashtra is worse than some of the world’s poorest countries such as Bangladesh (33% underweight), Afghanistan (25% underweight) or Mozambique (15%) [5–7].

**Fig 1:**
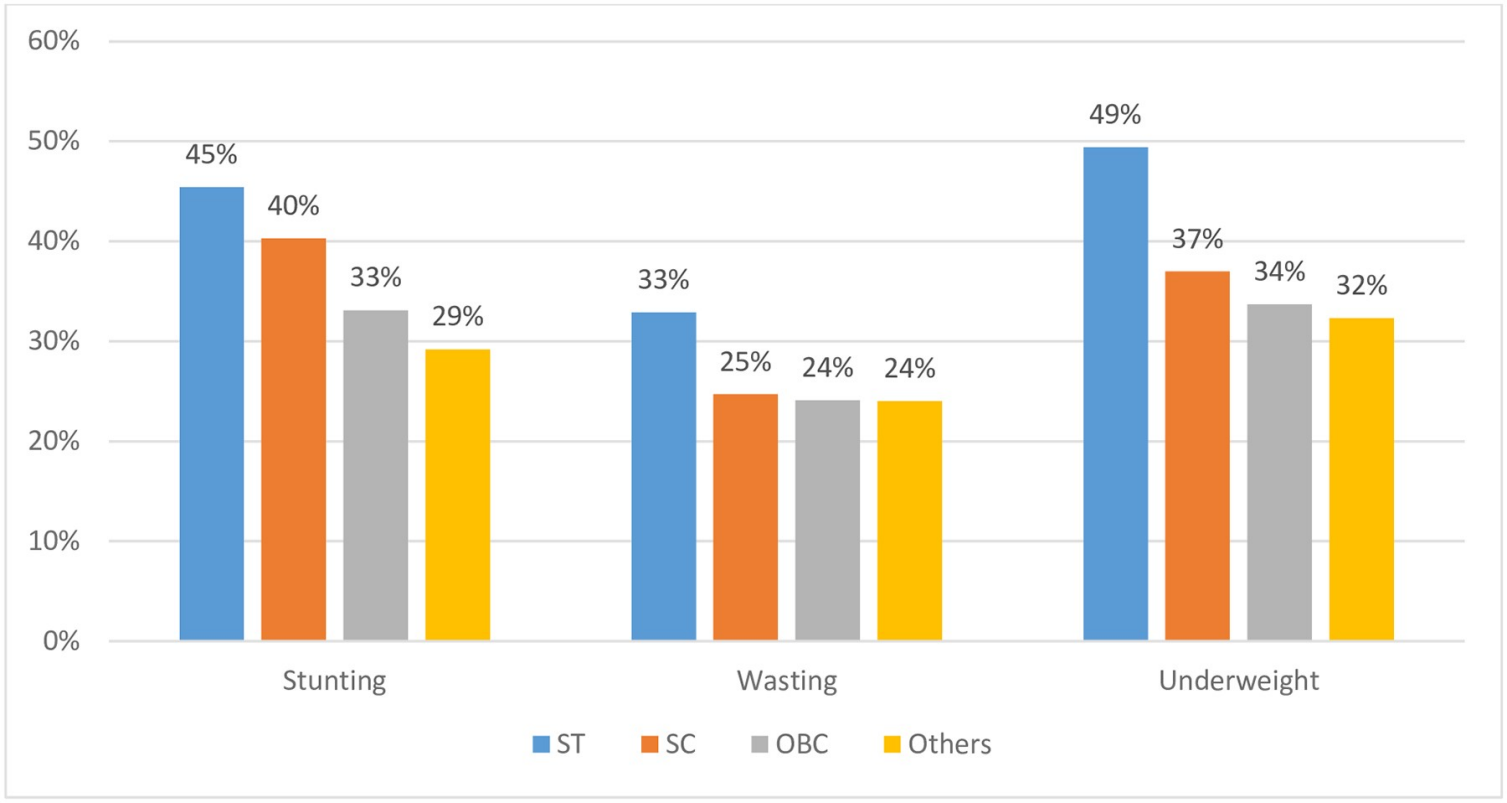
Nutritional status of children aged 0–5 years, Maharashtra, 2015–16. Source: NFHS 2015–16.

This level of poor nutrition security in Maharashtra disproportionately affects the poorest segment of the population in the state. Scheduled Tribes (ST) are one of the most disadvantaged social groups in Maharashtra, who suffer from perpetual food insecurity [8]. As per 2011 Census, 10.5 million tribal people are living in Maharashtra [9]. Loss of traditional forest dependent livelihood, unemployment and migration to cities such as Mumbai, Thane and Nashik and Gujarat to work as daily wage earners often employed in unskilled, exploitative jobs reduces the chances of the tribal households being able to crawl out of destitution. Within Maharashtra, all parameters of undernutrition-stunting, wasting and underweight are highest among the tribal children with almost half of tribal children under five years of age being stunted (Fig 1).

This is the worst form of malnutrition which cannot be easily reversed. Globally, stunting is responsible for nearly a third of the deaths of children below five [10]. In the tribal dominated districts of Maharashtra, child deaths due to malnutrition has become a regular phenomenon. In 2005, child malnutrition claimed as many as 718 lives in one single district namely Palghar (this was earlier part of Thane district). Interestingly, even though the state has recorded a decade of double digit economic growth between 2004–05 and 2014–15 (On average, GDP growth rate was 11.1% during this period), the nutritional status of the tribal children in Palghar has barely improved. In September 2016, the National Human Rights Commission issued a notice to the Maharashtra government over reports of 600 children dying due to malnutrition in Palghar district. It created a lot of hue and cry nationwide after media broke the story of continuing malnutrition deaths in Maharashtra. In response, aside from promising to properly implement schemes like APJ Abdul Kalam Amrut Yojana and Integrated Child Development Scheme (ICDS), the state government decided to reopen village child development centres (VCDC) in tribal pockets of Palghar to address malnutrition. APJ Abdul Kalam Amrut Yojana launched in 2015 by the Tribal development department of Maharashtra Government aims at providing at least one nutritious meal to pregnant and lactating women at the Anganwadi centre as per the convenience of the beneficiaries. However, to the best of our knowledge, there has not been any study to check whether the nutritional scenario has improved since then in Palghar.

Against this backdrop, the present study was undertaken with specific objectives such as, to assess the magnitude of under-nutrition among children under six years of age in the Vikramgad block which is predominantly a tribal area in Palghar District, to understand the causes of under-nutrition amongst the children and to examine the dietary pattern and food practices among the tribal population.

## Materials and methods

### Study area

Palghar was carved out of Thane district on 1st August 2014. Total population of Palghar was about 3 million in 2011 with 37.4% of Palghar’s population made up of STs. There were 1.37 lakh people living in Vikramgad, of which 1.26 lakh or 91.82% was ST population. It may be noted that Vikramgad block is a scheduled area as majority of the inhabitants of this block are tribals. Warli, Katkari, Malhar koli are the tribal groups residing in Palghar District. The district comprises of 8 talukas, namely Mokhada, Talasari, Vasai, Vikramgad, Jawhar, Palghar, Dahanu and Wada. There are a large number of tribal villages and hamlets in the hilly forested regions and a number of tiny fishing villages. Mokhada and Jowhar talukas were frequently mentioned in news and subsequently witnessed frenzied visits by the concerned government officials. The situation in Vikramgad Taluka was similar with nearly 30% of the children aged between 0–5 years being underweight as per the programme data of ICDS [11]. Hence, it was chosen as the study area. According to Census 2011, the male and female literacy rate was 53.60% and 46.27% respectively. Vikramgad has 94 villages and 521 padas. The word ‘pada’ indicates a hamlet inhabited by a particular tribe. These hamlets are scattered all over the area with some situated in very remote locations where accessibility is an issue, especially in the rainy season. The main occupation of the people here is farming, primarily rain-fed cultivation and hence, crops are grown only in one season. The crops grown are chiefly rice and finger millet. Finger millet or nachni is a nutritious cereal rich in calcium, iron and proteins and yet malnutrition is rampant in this region.

### Ethical statement

The study protocol was approved by the Board of Studies, School of Health Systems Studies, Tata Institute of Social Sciences, Mumbai. The respondents were explained about the objectives of the study and were provided with all the information about research, its outcome and implication of finding and they were encouraged to ask for any clarifications. Written informed consent was taken from the respondents on the informed consent form which was also printed in Marathi and was read out for women who were illiterate. They were assured that their privacy would be respected, and confidentiality would be maintained. Participants were allowed to withdraw from the study at any stage.

### Sampling

The sample size was calculated by employing a formula for estimation of a single proportion according to the following assumptions: 30% of under-five children are underweight [10], with 95% confidence interval and 5% margin of error. The calculated sample size was 317. A design effect of 1.2 was applied. Hence, the final sample size was 381. The sample was selected through a two-stage stratified random sampling design with villages as the primary sampling unit (PSU) at the first stage (Probability proportional to size method was used), followed by a random selection of 20 households in each PSU at the second stage. The inclusion criterion for selection of households was that the households must have at least one child between one and six years of age. So, households with children between one to six years of age whose mothers were available and willing to be interviewed were included in the sample. The survey was carried out during April-June 2017. While the height of the children was measured with an inch measuring tape, their weight was taken with the help of a weighing machine. Weight was recorded to the nearest 0.5 kg. All the participants (older than 24 months) were required to wear only light clothing and stand erect, barefoot and at ease while being measured. Children younger than 24 months were measured lying down. Children who were unable to stand or aged below 2 years were weighed along with their mothers and then mothers were weighed alone to derive the child’s weight by taking the difference of the two. Both the tape and weighing scale were calibrated before use and the same instruments were used in measurement across villages. Apart from anthropometric data, we also collected information on feeding practices, socioeconomic and demographic characteristics of the study population. A diet survey was also undertaken by following a 24-hour recall method. The schedule was administered to the mother. She was asked to recall what food was consumed by the child the day before the survey date starting from the most recent meal and going backwards until 24 hours were complete.

Apart from primary data, we have also utilised budget data for various years to understand the trend in public expenditure on nutrition.

### Method

The assessment of the nutritional status of the children was carried out using the 2006 WHO Child Growth Standard [12]. The height, weight and the age of the children were entered into WHO Anthro program for generating *z*-scores. Children whose anthropometric measures were below 2 Standard Deviation (SD) values of the WHO median reference (<median– 2 SD) based on ‘height-for-age’, ‘weight for- height’ and ‘weight-for-age’ indices were classified as having stunting, wasting and under-weight respectively. The children with measures ranging between median -3 SD and median -2SD were categorised as moderately malnourished while those with z-scores below 3 SD values of the reference median (<median– 3 SD) were defined as severely malnourished.

Aside from calculating the prevalence of malnutrition by age and sex, bivariate analysis and multivariate logistic regression models were undertaken to understand the independent effects of predictor variables on stunting, wasting and underweight.

#### Dependent variables

Stunting (height for age), wasting (weight for height) and underweight (weight for age) for children aged between 1 and 6 years. The dichotomous variables stunting, wasting and underweight were defined as 1 = stunted, else = 0; 1 = wasted, else = 0 and 1 = underweight, else = 0.

#### Explanatory variables

Variables were selected based on their effects on malnutrition.

**Sociodemographic variables**: Age and sex of the children, household size, birth order, type of house (whether living in a Kuccha or Pucca house), birth interval, maternal education, age at marriage for the mother of the child, age at first pregnancy and type of Tribe. Age has been grouped into following categories: 1–2 years, 2–3 years, 3–4 years, 4–5 years and 5–6 years.

#### Child health, feeding practices and utilisation of ICDS services

Health status of the children (whether the child fell sick in the past one month prior to the survey date), weaning age (age at which supplementary food was introduced), number of days the child went to Anganwadi centre and type of salt used.

Last but not the least, ***environmental variables*** such as whether child’s mother washes hands with soap, availability of latrine and source of water were also included.

In view of the very close correspondence between the findings of bivariate and multivariate analyses, and very little additional insights emerging from bivariate analysis, only results and discussions from multivariate analysis were retained. Only variables, that were found to be having statistically significant association with dependent variables in bivariate analyses, were included in the multivariate analyses. Chi-square tests were carried out to test the association between independent and dependent variables. All analyses were performed using STATA 14 software.

A critical aspect of nutrient adequacy is diet diversity. Food eaten by the children during the last 24 hours before the survey date was classified into the following eight food groups: (1) Cereals, roots and tubers (2) Legumes and nuts (3) Dairy products (4) Flesh foods (5) Eggs (6) Fish (7) Dark green leafy vegetables (8) Fruits and other vegetables. We calculated a 24-hour dietary diversity score (DDS) by counting the number of food groups the child received in last 24-hours prior to the survey date. A DDS of four is considered the minimum dietary diversity for children required to meet the daily energy and nutrient requirements. Children receiving foods from four or more groups are considered to have adequate dietary diversity.

## Results

### Characteristics of the study population

From the total of 381 planned study participants, complete response was obtained for 375 (98.4%). As shown in Table 1, a total of 166 male (44%) and 209 female (56%) children were involved in this study. The mean age of the male and female children was 2.93 and 3.18 years respectively. Among the 375 children covered in the survey, about 37% were the first born children, 50% were of either of second or third order, while 13% were of fourth or higher order. Almost a fourth of the children were born within 2 years of their elder sibling while one-third had a birth interval of 2–3 years. Of the surveyed children, only 44% were born with an interval of more than three years which is considered to be ideal for the health of the child as well as mother.

**Table 1:** Background characteristics of the tribal children 1–6 years under study, Palghar district, Maharashtra, 2017.

Background characteristics	N = 375	%
**Age (years)**		
1–2	89	23.7
2–3	78	20.8
3–4	70	18.7
4–5	78	20.8
5–6	60	16.0
**Gender**		
Female	209	55.7
Male	166	44.3
**Education (mother of the child)**		
Illiterate	178	47.5
Primary	52	13.9
Secondary	145	38.6
**Age at marriage (mother of the child)**		
< 18 years	185	49.6
18–20 years	165	44.2
21 yrs & above	23	6.2
**Age at first pregnancy (mother of the child)**		
< 18 years	85	22.7
18–20 years	201	53.6
21 yrs & above	89	23.7
**Birth order of child**		
1	140	37.3
2 to 3	186	49.6
4 or more	49	13.1
**Birth interval**		
Less than 2 years	55	23.6
2–3 years	76	32.6
More than 3 years	102	43.8
**Household size**		
Up to 5	127	33.9
6–7	164	43.7
8+	84	22.4
**Source of drinking water**		
Improved water supply	174	46.4
Unimproved water supply	201	53.6
**Toilet facility**		
**Yes**	230	61.3
No	145	38.7
**Type of salt used**		
Iodised	141	37.6
Non-iodised	234	62.4
**Type of house**		
Kuccha	93	24.8
Semi pucca	282	69.9
Pucca	20	5.33
**Tribe**		
Malhar koli	82	21.9
Warli	247	65.9
Others	46	12.3

Majority of the children’s mothers were in the age group of 21 to 30 years, with the mean age being 26.9 years. Nearly half (47.5%) of the children’s mothers interviewed were illiterate. About 13.9% had primary education; 36% had secondary education while only about 2.7% had higher secondary education. The information collected on the age at marriage of the children’s mothers revealed that one in every two became married even before they attained the legal age at marriage i.e., 18 years. Moreover, almost one-fourth of the women were pregnant before the age of 18 years and 53.6% had their first pregnancy between their 18^th^ and 20^th^ birthday. Only 23.7% of the children’s mothers were above 21 years of age at the time of their first pregnancy.

Majority of the children belonged to the Warli tribe (66%), followed by Malhar Koli (22%), Konkona, Katkari and K Thakur tribes (12%). About 60% of the sampled children lived in joint families. The average family size was 6.4 and ranged from a minimum of 3 to a maximum of 16 members in a family. The living conditions of the families with children under study can be understood from the fact that 95% of them were found to be living in Kuccha (25%) and Semi-pucca houses (70%). Further, almost 40% of the households did not have toilet and continued to practise open defecation; nearly 9 out of 10 families used wood as fuel for cooking. They collected firewood from forests and carried it back home on their heads.

More than half of the households reported to use well-water (54%), which is considered as an unimproved source of water according to WASH guidelines [13] whereas, 46% of the households got water from an improved source such as a hand-pump or bore-well, that are usually provided by the gram panchayat. Also, a few households (11.7%) got piped water in their houses which was seen in certain villages where the water from the bore-well was stored in an overhead tank and then distributed to the households in the vicinity. Only two of the sampled households said that they used boiling as a method of water treatment for drinking. Further, the use of iodised salt was also very less with only 38% of the households using iodised salt and of which about 7% were using both iodised and non-iodised types. Majority of the households (62%) still used non-iodised salt as non-iodised salt is less expensive compared to the iodised salt.

### Nutritional status of children between 1–6 years

**Fig 2:**
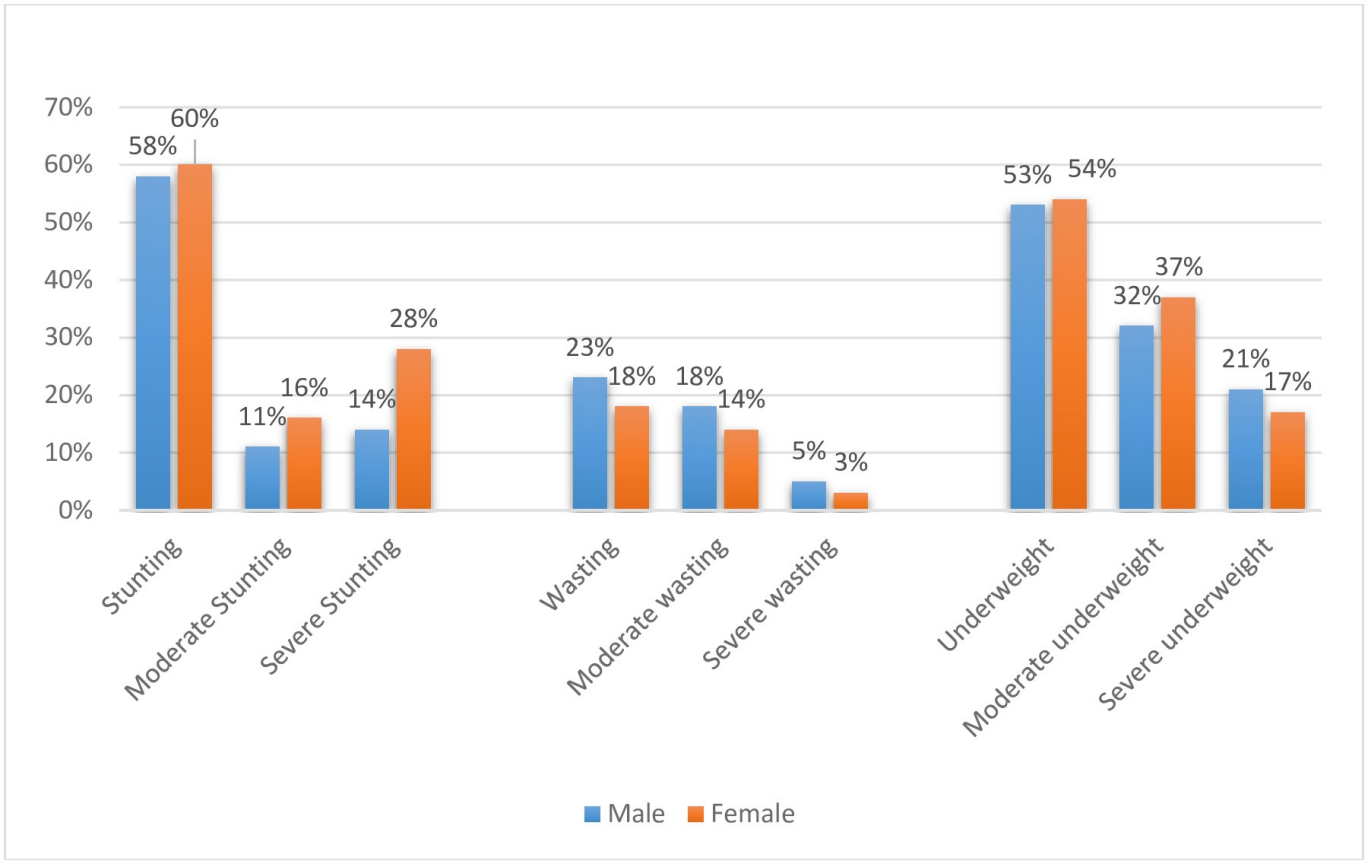
Prevalence of stunting, wasting and underweight among the children by sex, Vikramgad, Maharashtra, 2017.

**Fig 3:**
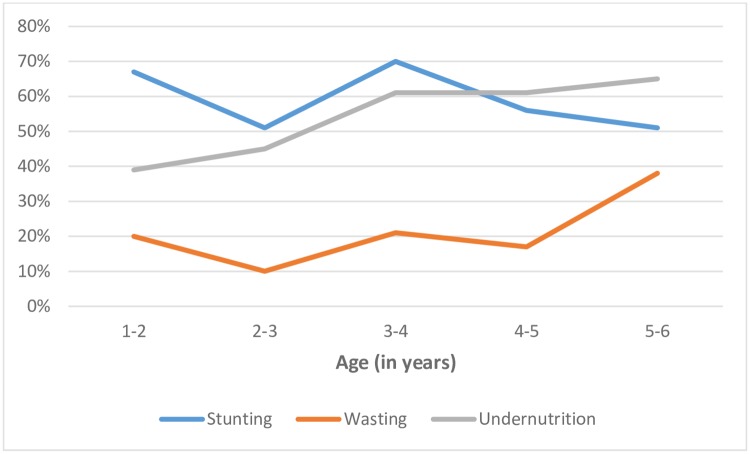
Prevalence of stunting, wasting and underweight among the children by age, Vikramgad, Maharashtra, 2017.

The results indicate that 59% of children were stunted; 32% were moderately stunted and 27% had severe stunting. The overall prevalence of wasting was 20% and the prevalence of severe wasting was 4%. Further, the prevalence of underweight was about 53% and prevalence of severe underweight was 18%. The prevalence of stunting and underweight was similar across sexes, though wasting was considerably higher amongst the male children compared to their female counterparts (Fig 2). When examined by age, no discernible pattern was observed in case of prevalence of stunting, though it was found to be highest among the children aged between 3 and 4 years (Fig 3). On the other hand, wasting showed a declining trend until the age of 3 and then it increased sharply. The most consistent pattern was noticed for underweight, which steadily increased with age.

### Factors associated with stunting, wasting and undernutrition

Table 2 shows the results of the multivariate logistic regression on stunting. The analysis suggests that older children were less likely to be stunted compared to those aged between 1 and 2 years. Children for whom weaning was started at the age of 10 months or more compared to those for whom weaning was started at the age of 6 months or below were found to be significantly less likely to be stunted (OR = 0.39; p<0.02). Children living in larger households with 6 to 7 and 8 or more members had significantly greater odds of being stunted than children residing in households up to 5 members (OR = 1.7, p<0.04; OR = 1.9, p<0.04). The results also revealed that children from the Konkona, Katkari and K Thakur tribes were at a significantly higher risk of becoming stunted than their counterparts from Malhar tribe (OR = 2.38). According to the results of the multivariate analysis, age was found to be associated with wasting (Table 3). Children aged between 5 and 6 years were 2.5 times more likely to be wasted than children who were 1 or 2 year/s old (OR = 2.49; p<0.02). Table 4 shows the findings of the logistic regression on underweight. The results indicate a positive relationship between age and underweight.

**Table 2:** Results of multivariate logistic regression analysis of stunting in tribal children 1–6 years, Vikramgad, Maharashtra, 2017.

characteristics	categories	Odds ratio	Lower	Upper	P>z
**Age group (years)**	1–2	1			
	2–3	0.43	0.22	0.84	0.014
	3–4	0.91	0.44	1.88	0.807
	4–5	0.46	0.23	0.92	0.020
	5–6	0.47	0.13	0.76	0.040
**Weaning age**	By 6 months	1			
	7–9 months	1.53	0.41	1.56	0.134
	10 or more	0.39	0.18	0.88	0.023
**Household size**	Up to 5	1			
	6–7	1.70	1.02	2.83	0.042
	8 or more	1.90	1.02	3.55	0.044
**Tribe**	Malhar	1			
	Warli	1.31	0.68	2.37	0.343
	Others	2.38	1.05	5.35	0.037
Log likelihood = -229.330			Pseudo R2 = 0.0635		
LR chi2(23) = 36.59			Prob > chi2 = 0.0011		

**Table 3:** Results of multivariate logistic regression of wasting in children 1–6 years, Palghar district, Maharashtra, 2017.

characteristics	categories	Odds ratio	Lower	Upper	P>z
**Age group (years)**	1–2	1			
	2–3	0.46	0.18	1.14	0.11
	3–4	1.09	0.49	2.41	0.74
	4–5	0.82	0.37	1.85	0.64
	5–6	2.49	1.17	5.29	0.02
Log likelihood = -176.91				LR chi2(23)	32.82
Prob > chi2	0.0024			Pseudo R2	0.0445

**Table 4:** Results of multivariate logistic regression of underweight in children 1–6 years, Palghar district, Maharashtra, 2017.

characteristics	categories	Odds ratio	Lower	Upper	P>z
**Age group (years)**	1–2	1			
	2–3	1.36	0.71	2.58	0.352
	3–4	2.46	1.37	5.97	0.008
	4–5	2.72	1.17	4.96	0.003
	5–6	3.28	1.61	6.67	0.001
**Household size**	Up to 5	1			
	6 to 7	2.27	1.38	3.73	0.001
	8 or more	1.62	0.91	2.89	0.103
**Health status**	whether sick				
	No	0.48	0.28	0.80	0.005
Log likelihood = —237.08299			LR chi2(23)	=	40.97
Pseudo R2	= 0.0795		Prob > chi2	=	0.0083

In other words, the risk of being underweight was found to be significantly rising with the increase in age. Also, according to the findings, children from households with 6 to 7 members were 2.3 times more likely to be underweight than those from smaller households. Children, who did not suffer from any illness in the last 30 days prior to the survey date, had significantly lower likelihood of being underweight than the children who happened to be sick during the same period (OR = 0.48; p<0.005).

### Dietary diversity

**Fig 4:**
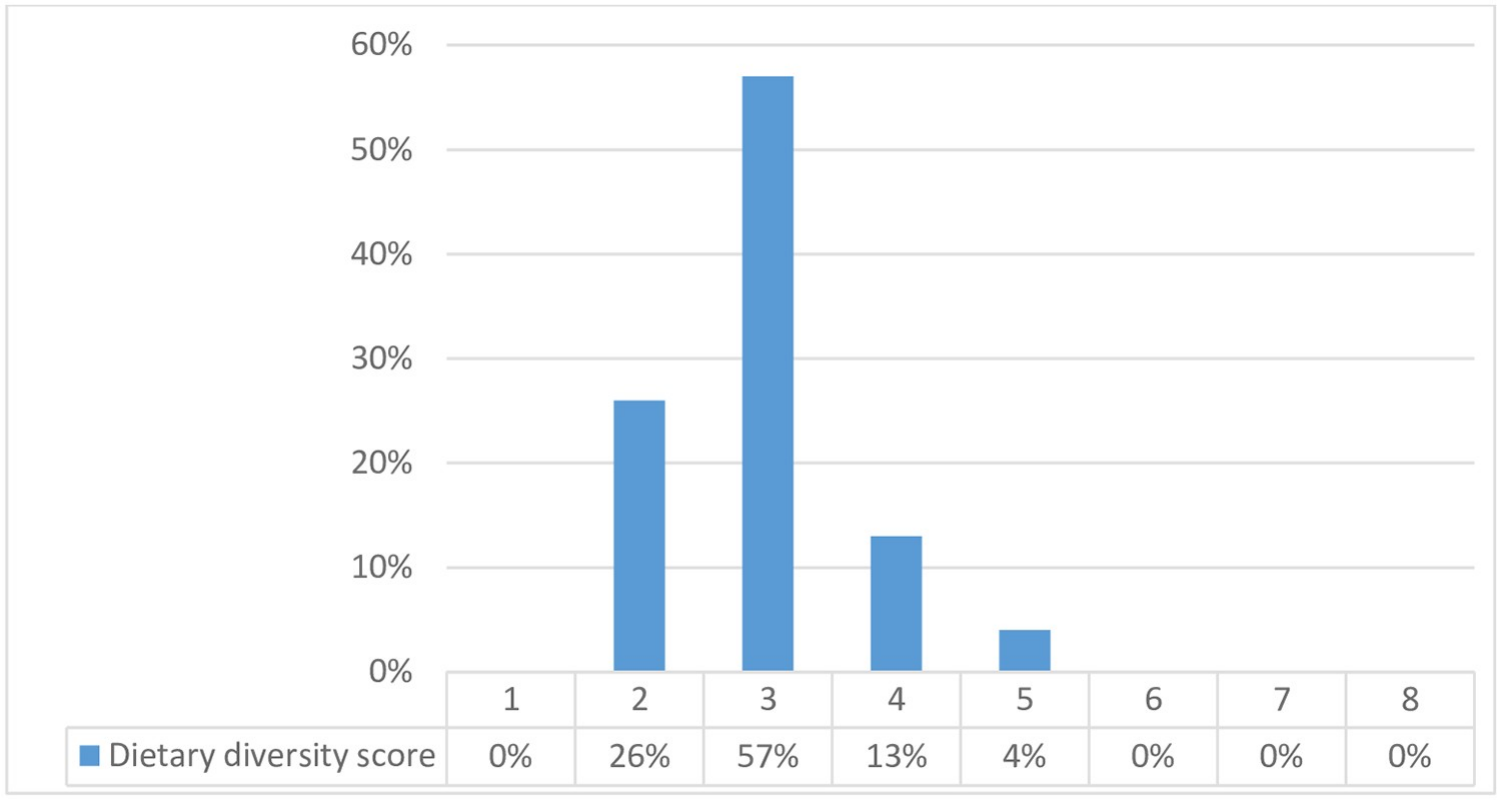
Dietary diversity score for tribal children, Vikramgad, Palghar district, 2017.

As shown in Fig 4, 26% of the children had a Dietary Diversity Score (DDS) of 2 which means they had received food from only two of the listed food groups. Nearly 57% of children had a DDS of 3. This means that 83% of the children did not have an acceptable minimum diet. The most common food eaten was rice and dal. In many households, it was this combination that was cooked for all the three meals of the day. The same was served even at teatime to the children if they felt hungry. Milk and milk products and Fruits and vegetables were conspicuously absent in their diets. Even the adults drank black tea as milk was unaffordable. Only 13% of the children achieved a minimum level of diet diversity i.e., they received four or more of eight food groups.

**Fig 5:**
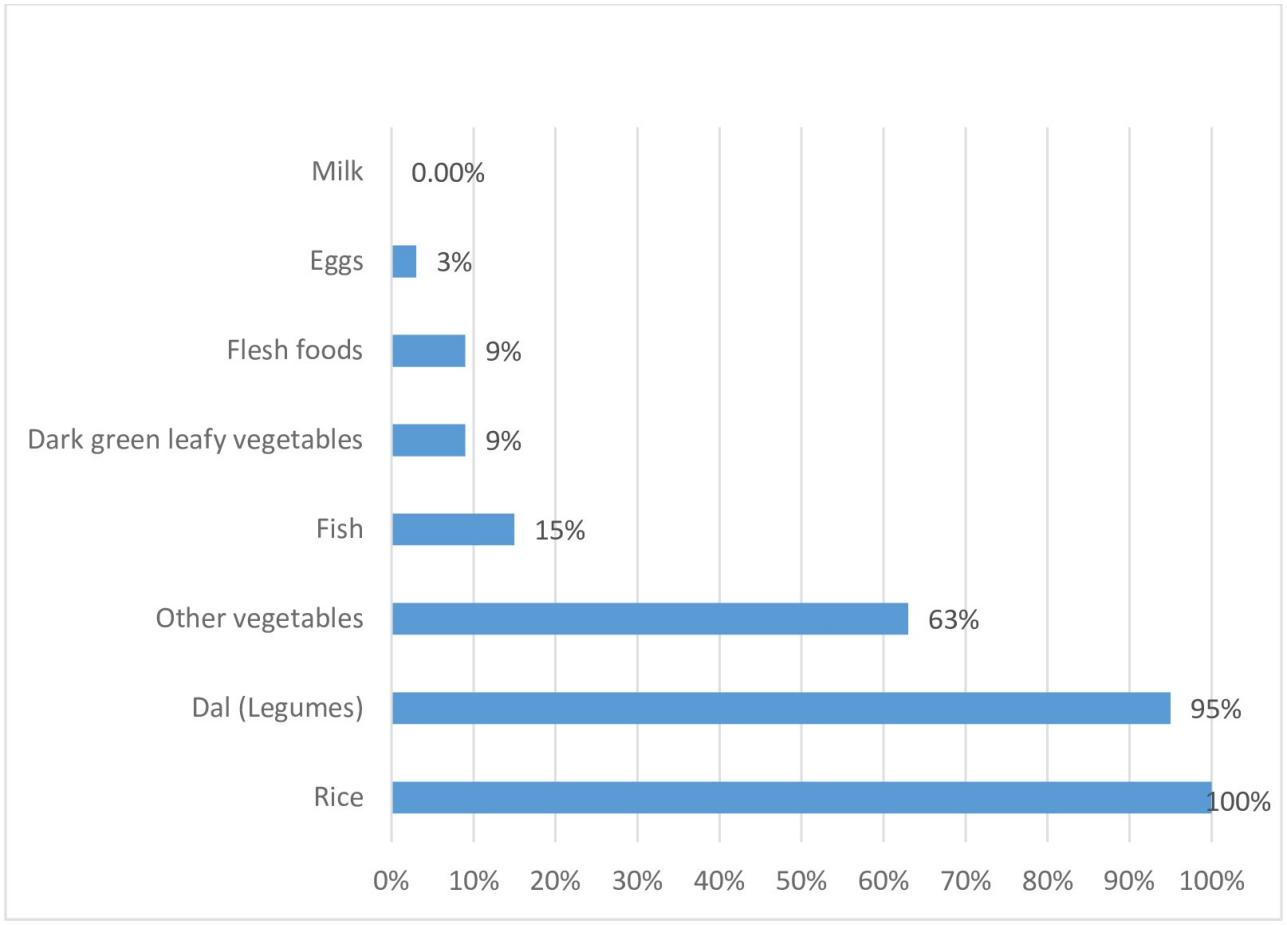
Consumption of each of the food groups by the tribal children in Vikramgad, Palghar district, 2017 (in percent).

In order to get further insights into the diet diversity of the tribal children in Palghar, the percent consumption of each of the eight food groups was estimated (Fig 5). The proportion of children who had eaten rice and dal (legumes) were 100 percent and 95 percent respectively. The next most common food group consumed by the children was ‘other vegetables’ (63%), which included lady’s finger, egg-plant, drumstick and gavar (cluster bean). Flesh foods and fish were consumed by 9% and 15% of the children. Milk was reported to be consumed by only one child and eggs were eaten by only 3% of the children. The consumption of green leafy vegetables, which is a good source of iron, was also very rare and only 9% of the children ate leafy vegetables.

## Discussion

Our study level estimates suggest that the prevalence of undernutrition among the tribal children aged between 1 and 6 years remained alarmingly high in Palghar district. The proportion of children with stunting, wasting and underweight was 59%, 20% and 53% respectively in 2017. In the current study, the prevalence of under-nutrition was found to be associated with children’s age, their health status, family size and ethnicity. The older children seemed to be at a relatively lower risk of stunting, but they were found to have higher odds of wasting and underweight than the younger children. Children who were weaned much later (10 months or more) than the recommended age of 4 to 6 months were significantly less likely to be stunted. This implies that despite weaning at the recommended age, those children did not receive the necessary nutrition through the complementary feeds and also missed out on the nutrition available through breast milk. Having sickness episode was another risk factor of under-nutrition. Children who did not suffer from any disease like diarrhoea, cold or any other acute illness were significantly less likely to be underweight. The result was consistent with previous studies that malnourished children would be more prone to sickness or sick children would tend to be underweight, thus confirming the two way relationship between undernutrition and health [14, 15]. Children living in larger households with more than 6 members (and hence, most likely to be in multigenerational households) were more likely to be stunted or underweight than those from smaller families with not more than 5 members. A high prevalence of stunting among the children living in larger households was also documented by other studies [16]. Inadequate availability of food and inequality in the intra-household distribution of food may possibly explain the reason behind the higher prevalence of undernutrition in larger households. Children from the Konkona, Katkari and K Thakur tribes are most vulnerable and possibly, food insecure amongst all tribes under study. They were at higher risk of stunting than their counterparts from Malhar tribe.

There is a growing body of literature that suggests early childhood malnutrition is fundamentally linked to poverty and food insecurity. Our study findings are clearly in agreement with the existing literature [17–19]. The food consumption pattern observed in our sample of children was characterised by almost zero intake of leafy vegetables, fruits, milk and milk products, flesh food, fish and eggs. The sample children’s diet was composed mainly of rice and dal. Thus, they could not achieve a minimum level of diet diversity, which is critical for their growth. The consumption patterns of children actually reflected the eating patterns of their families. Such acute food insecurity in tribal households is due to the loss of their traditional dependence on forest livelihood and the state’s deepening agrarian crisis [8, 20]. Besides loss of livelihood, systemic issues such as exclusions in public distribution system and weakening of public nutrition programmes have aggravated the undernutrition problem. In our sample, one-fifth of the tribal families did not receive ration through public distribution system in Vikramgad due to non-possession of the card. These findings are corroborated by past studies that linked the continuing nutrition deprivation in tribal children to poor access to food and nutrition entitlements [21, 22].

## Conclusion

The present study confirms that despite several nutrition programmes, the extent of undernutrition has remained extremely high in the children living in predominantly tribal rural areas of Palghar district, Maharashtra. One of the possible reasons for having persistently high level of undernutrition among tribal children is the decline in the budgetary allocation on ICDS in recent years [20]. Our analysis of the state’s budget also reveals that the nutrition expenditure as a percentage of the state budget has drastically declined from 1.68% in 2012–13 to 0.94% in 2018–19 (S1 Fig). Moreover, Singh and Sethi [22] showed that tribal sub plan funds have been underutilised by Maharashtra, particularly in two sectors (rural development and nutrition). As the main underlying cause of undernutrition among the tribal children is the poor socioeconomic conditions of the tribal population, there is a need for a multi-pronged strategy for redressing this problem.

Aside from increasing the budgetary allocation for nutrition specific interventions such as APJ Abdul Kalam Amrut Aahar Yojana and ICDS, the state needs to ensure appropriate usage of funds. In addition, there is an urgent need to tighten the implementation mechanism of the Public Distribution System (PDS) in the tribal areas of Palghar. The department of Civil Supplies (food security) must ensure that tribal families are not deprived of the ration that they are entitled to because of issues like non-possession of card. Besides that, Maharashtra should seriously consider providing other nutritious food items through PDS in the tribal areas so that the nutritional needs of the tribal children and adults are met, and the problem of micronutrient deficiencies is effectively addressed.

Aside from improving socioeconomic conditions of the tribal population, there is a need to improve the child care and feeding practices. The positive breast-feeding practices related to exclusive breastfeeding during the first half of infancy and continued breastfeeding through the second year of life and beyond encountered in the population should be strongly promoted. Practices that require attention are the delay in introduction of complementary foods and the low dietary diversity. This should be addressed by providing education about child care and feeding practices to the women from the time they become pregnant.

## Supporting information

S1 Fig (PDF)

S1 Tool (PDF)

S2 Tool (DOCX)

S1 Data (RAR)
