# Supplementary material for: Undernutrition among tribal children in Palghar district, Maharashtra, India
Source: PLoS One. 2019 Feb 27;14(2):e0212560. doi: 10.1371/journal.pone.0212560 (PMC6392283; doi:10.1371/journal.pone.0212560)

S1 Appendix

S1 Fig. Trends in government expenditure on nutrition in Maharashtra (in nominal terms)

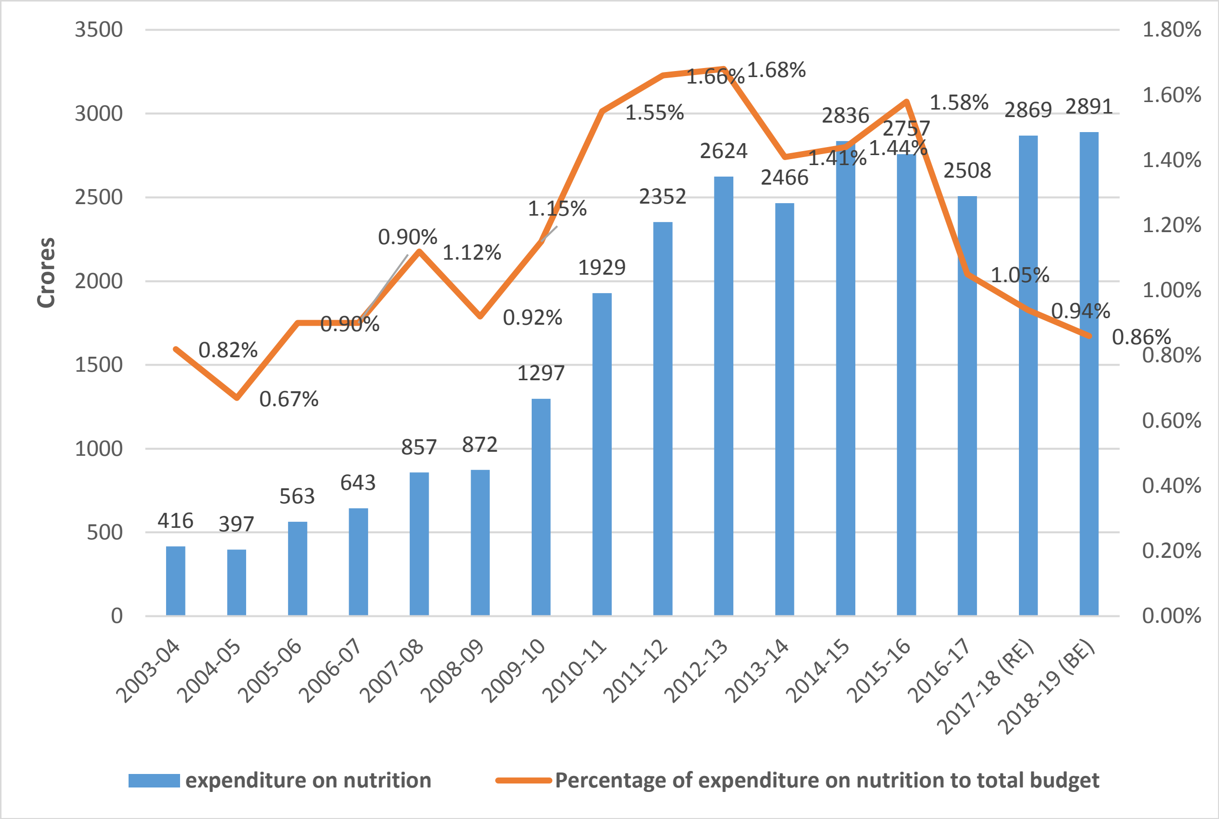

Supplement: S1 Fig — (PDF) [file pone.0212560.s001.pdf]
