# Supplementary material for: Undernutrition among tribal children in Palghar district, Maharashtra, India
Source: PLoS One. 2019 Feb 27;14(2):e0212560. doi: 10.1371/journal.pone.0212560 (PMC6392283; doi:10.1371/journal.pone.0212560)
Supplement: S1 Tool — (PDF) [file pone.0212560.s002.pdf]

दिनांक	PHC	AWC	उपकेंद्र	गाव	पाडा	

शरीरासंबंधी माहिती

क्र.	बाळाचे नाव	वय	लिंग	गर्भधारणेनंतर कोणत्या महिन्यात प्रसुती झाली	बाळाचे वजन	बाळाची उंची	जन्मक्रम

९. सामाजिक व आर्थिक संबंधी माहिती

९.१	जमात	१. महादेव कोळी	२. कातकरी	३. वारली	४. इतर	
९.२	घराचा प्रकार	१. कच्चा	२. साधारण पक्के	३. पक्के		
९.३	घरात लाईट आहे का?	१. वीज	२. कंदिल	३. इतर		
९.४	शौचालयाची सोय	१. घरात शौचालय	२. सार्वजनिक	३. शेतात		
९.५	पाणी पिण्याची सोय	१. पाईपद्वारा पाणी	२. विहिर	३. हात पंप	४. तलाव	५. इतर
९.६	पाणी कसे पितात ?	१. गाळुन – कपड्याने /फिल्टर		२. उकळुन	३. तुरटी	५. इतर
९.७	आयोडिनयुक्त मीठ वापरतात	१. हो	२. नाही			
९.८	जेवण कशावर बनवतात	१. लाकुड	२. घासलेट स्टोव्ह	३.गॅस	४. शेणी	५. इतर
९.९	जेवण कुठे बनवतात	१. स्वयंपाकघर	२. अंगण	३. एकच खोली		
९.१०	आईच्या हात धुवायच्या पद्धती	१. साबण	२. राख	३. फक्त पाणी	४. इतर	
९.११	मुलाच्या हात धुवायच्या पद्धती	१. साबण	२. राख	३. फक्त पाणी	४. इतर	
९.१२	तुम्ही गाव सोडून कामासाठी स्थलांतर करता का? गेलात तर, किती दिवसांसाठी?	१. हो	२. नाही			
९.१३	मागील वर्षी तुम्ही स्थलांतर केले होते का?	१. हो	२. नाही			
९.१४	गेल्या ५ वर्षात तुम्ही किती वेळा स्थलांतर केले ?					
९.१५	पूर्ण कुटुंबासहित तुम्ही स्थलांतर करता का?		१. हो	२. नाही		
९.१६	जेव्हा तुम्ही स्थलांतर करता तेव्हा तुम्हाला सरकारी योजनेचा काही फायदा झाला आहे का?				१. हो	२. नाही
९.१७	जेव्हा तुम्ही स्थलांतर करता तेव्हा मुलांना लस टोचता का?					
९.१८	तुमचे मुल कुपोषित होते का? केव्हा?		१. हो	२. नाही		

कुटुंबाविषयी माहिती

१०. कुटुंबात किती माणस आहेत						
क्र.	नाव	वय	लिंग पु./स्त्री	कुटुंबप्रमुखाशी नात	शिक्षण (किती वर्षे शाळेत होता)	व्यवसाय

१. स्वतःच्या शेतामध्ये काम करतात
२. शेती–व्यतिरिक्त स्वतःचा व्यवसाय
३. दुसऱ्यांच्या शेतात मजुर म्हणून काम करतात
४. मजुर म्हणून दुसरीकडे काम करतात
५. पगारी काम दुसरीकडे काम करतात
६. बेरोजगार आहात

घरात रोजच्या वापरात येणाऱ्या अन्नधान्य आणि इतर लागणाऱ्या गोष्टींचा खर्च व त्यासंबंधी जमा केलेली माहिती.

उपयोगात येणाऱ्या गोष्टी / खर्च

११	घरात महिन्याला लागणारे सामान	तुम्ही विकत घेता १. हो २. नाही	किती प्रमाणात	विकत घेत असाल तर किती पैसे खर्च करता	घरी पिकवता का ? १.हो २. नाही	किती	किंमत	तुम्ही ते उसने घेतले का?	कशाच्या बदल्यात घेतले का?
	तांदुळ – रेशनिंग								
	तांदुळ – इतर								
	गहु – रेशनिंग								
	गहु – इतर								
	कडधान्य								
	डाळी								
	कांदे								
	बटाटा आणि कंद								
	हिरव्या भाज्या								
	दुध–दहि								
	मांसाहारी								
	फळे–सुकामेवा								
	मसाला								
	मीठ–साखर								
	गॅस–लाकूड–घासलेट								
	प्रसाधनवस्तू								
	वीज								
	वाहतूक								
	मोबाईल								
	पान–विडी–सुपारी								
	करमणुक								
	सण–कार्यक्रम								
	आरोग्यकेंद्र								

	सामान	गेल्या वर्षी घराला लागणारे सामान विकत आणले<br>१. हो २. नाही	जर हो,<br>किती पैसे वापरले
	गादी आणि चादर		
	कपडे आणि चप्पला		
	शाळेची फी आणि पुस्तके		
	घरातल्या वस्तू		
	स्वयंपाकघरातील वस्तू		
	दागिने		
	मोठी दुरुस्ती		
	विमा		
	आरोग्य – IPD		

१२. स्तनपान

			मुल – १	मुल – २
१२.१	दोन प्रसुती मधील अंतर			
१२.२	बाळाच्या जन्मानंतर किती वेळानंतर स्तनपान केले	१. लगेच २. अर्ध्यातासानंतर<br>३. २४ तासानंतर<br>४. २४ तासापेक्षा जास्त		
१२.३	जर नाही, तर काय दिले			
३४.	बाळाला पहिले स्तनपान केले का?	१. हो २. नाही		
३५	सहा महिन्यांपर्यंत फक्त स्तनपान केले का?	१. हो. २. नाही		
३६	जर नाही तर काय कारण			
३७.	स्तनपान दिवसातून किती वेळा करतात			
३८.	किती दिवसापर्यत स्तनपान केले			
३९.	तुम्हाला स्तनपानाबद्दल माहिती होती का ? कुणाकडुन?	१. अंगणवाडी ४. नर्स<br>२. आशा ५. डॉक्टर<br>३. दाई ६. इतर		

१२. आहार

४०	वरचे खाणे कधीपासून दिले			
४१	उशीराचे कारण काय			
४२.	कुठल्या प्रकारचे जेवण देता	१. पातळ – भाताची पेज, डाळीचे पाणी, दुध, इतर<br>२. थोडे जाडसर – कांजी, खिमटी<br>३. अन्न – वरण–भात, भाकरी– भाजी, अंडे, इतर		
	तुम्ही जे अन्न खाता तेच लहान बाळांना देता का?			

लसीकरण

		मुल – १	मुल – २
४३.	बाळाला लस टोचली आहे का? १. हो २. नाही		
४४.	जन्माच्या वेळी बीसीजी आणि पोलिओ<br>OPV1 + DPT 1 (६ आठवडे)<br>OPV2+ DPT2 (१० आठवडे)<br>OPV3+ DPT3 (१४ आठवडे)<br>Measles 1 (९–१२ म.)<br>Measles 2 (१६–२४ म.)		
४५.	व्हिटॅमिन A (९ महिन्यानंतर) १. हो. २. नाही		
४६.	जंताचा डोस १. हो २. नाही		
४७.	लसीकरण कुठे केले		

आजार

	जन्माच्या वेळी बाळ हॉस्पीटलमध्ये होते का?		
४८	गेल्या महिन्याभरात काही आजार झाला का?		
४९	झाल्यास, कोणता ? १. अतिसार २. ताप ३. सर्दी–खोकला		
५०.	असल्यास,बाळाला कुठे नेले ? १. आरोग्य केंद्र २. खाजगी डॉक्टर<br>३. भगत		
५१.	आजाराची लक्षण दिसल्यानंतर किती वेळात उपचार केले?<br>बाळाला दुसरीकडे कुठे नेले होते का?		
५२.	गेल्या वर्षभरात बाळाला हॉस्पीटमध्ये भरती केले होते का?		
५३.	बाळाला काही दीर्घ कालीत आजार आहे का?		

सामाजिक सुरक्षा योजनेचा वापर

५४.	गावामध्ये अंगणवाडी आहे का? १. हो २. नाही		
५५.	घरापासून अंगणवाडी किती लांब आहे?		
५६.	आपले मुल अंगणवाडीत जाते का? १. हो २. नाही		
५७.	तिथे अन्न दिले जाते का?<br>१. सुका खाऊ<br>२. घरी न्यायला खाणे<br>३. गरम खिचडी<br>४. फळ<br>५. अंडी		
५८.	अंगणवाडीत किती वेळा आरोग्य तपासणी केली जाते?<br>अंगणवाडीत किती वेळा मुलाचे वजन केले जाते?		
५९.	गेल्या महिन्यात किती दिवस मुल अंगणवाडीत गेले?<br>आजारपणामुळे अंगणवाडीत मुल गेले नाही का?<br>दुसऱ्या कोणत्या कारणाने अंगणवाडील मुल गेले नाही?		
६०.	सरकारी योजनेतून रेशन मिळते का?<br>तुम्हाला अंत्योदय अन्न योजनेचे कार्ड मिळाले का?		
६१	जननी सुरक्षा योजनेचा फायदा तुम्हाला मिळाला आहे का?<br>मातृत्व अनुदान योजनेचा फायदा तुम्हाला मिळाला आहे का?<br>महात्मा गांधी राष्ट्रीय ग्रामीण रोजगार योजना आणि रोजगार हमी योजनेतून तुम्हाला रोजगार मिळाला का?		

आता मी तुम्हाला तुमच्या शेवटच्या गर्भधारणेविषयी काही प्रश्न विचारते

आई विषयी काही माहिती

६२ वय	६३. लग्नाच्या वेळचे वय	६४. पहिल्या गर्भधारणे–वेळचे वय	६५. किती वेळा गरोदर राहिलात	६६. किती मुल आता हयात आहेत	६७. ए.एन.सी भेट	६८. आय. एफ.ए. गोळ्या घेतल्या	६९. टी.टी. इन्जक्शन	७०.प्रसुति पूर्व आरोग्य कसे होते	७१. प्रसुति कुठे झाली

आरोग्याविषयी मत : १. उत्कृष्ट २. खूप छान ३. छान ४. ठिक ५. ठिक नाही

७१.	अंगणवाडी तर्फे काही खास योजना दिल्या होत्या का?	१. हो २. नाही	
७२.	प्रसुती नंतर आरोग्याची तपासणी होते का?		१. हो २. नाही
७३.	आशा योजनेतर्फे गर्भधारणेविषयी माहिती मिळाली होती का?	१. हो २. नाही	
७४.	मुलांमधील अंतर<br>अ. पहिल्या आणि दुसऱ्या मुलामधले<br>ब. दुसऱ्या आणि तिसऱ्या मुलामधले<br>क. तिसऱ्या आणि चौथ्या मुलामधले		
७५.	कुटुंब नियोजनाची पद्धत १. गोळ्या २. कॉपर टी ३. कंडोम ४. टी.एल.		
७६.	काही व्यसन आहे का? १. तंबाखु २. दारु ३. इतर		

गेल्या २४ तासापूर्वी काय खाल्ले	पदार्थांची नाव	पदार्थात काय होते	किती अन्न खाल्ले	अन्नातील कॅलरी
नाश्ता	१.			
	२.			
	३.			
दुपारचे जेवण	१.			
	२.			
	३.			
संध्याकाळचा नाश्ता	१.			
	२.			
	३.			
रात्रीचे जेवण	१.			
	२.			
	३.			
इतर	१.<br>२.			

सहभागी लोकांची माहिती

प्रिय जन हो,

माझे नाव डॉ. सारिका वरेरकर. मी टाटा इन्स्टिट्यूट ऑफ सोशल सायन्सच्या पब्लिक हेल्थ डीग्री कोर्सची विद्यार्थिनी आहे. आमच्या पब्लिक हेल्थ डीग्री कोर्सच्या गरजेनुसार मी विक्रमगड तालुक्यातील आदिवासी मुलांमधील कुपोषणाच्या कारणाविषयी अभ्यास करीत आहे. मी तुमच्या कुंटुंबातील एका माणासाची घरगुती सर्वेक्षणा बद्दल मुलाखत घेणार आहे. तुमची घरगुती सर्वेक्षण माहिती गोळा करण्यासंबंधी मी खूप उत्सुक आहे.

मी तुम्हाला काही घरगुती प्रश्न विचारणार आहे. ही मुलाखत २० ते ३० मिनीटात पूर्ण होईल. तुम्ही जी काही माहिती द्याल ती पूर्णपणे गोपनीय ठेवण्यात येईल. तुमचे नाव, पत्ता आणि इतर माहिती पूर्णपणे गोपनीय ठेवीन याची मी हमी देते. तुम्ही दिलेली सर्व माहिती पूर्णपणे फक्त मुलांच्या कुपोषणांच्या कारणांवरील आदिवासी संशोधनासाठीच वापरली जाईल अन्यत्र त्याचा वापर होणार नाही.

ह्या सर्वेक्षणात सहभागी झालेल्या व्यक्तिला प्रश्नाचे उत्तर देणे हे ऐच्छिक राहिल. ह्या सर्वेक्षणात तुमचा सहभाग असणे फार महत्वाचा असल्यामुळे तुम्ही मला पूर्ण सहकार्य द्याल याची मला खात्री आहे.

ह्या वेळेला तुम्ही सर्वेक्षणाबद्दल प्रश्न विचारू शकता ?

मी आता तुमच्या मुलाखतीला सुरवात करु शकते का ?

संमतीपत्र

दिनांकः

विषय : विक्रमगड तालुक्यातील आदिवासी मुलांमधील कुपोषणाच्या कारणाबाबत

मी माझ्या इच्छेनुसार ह्या सर्वेक्षणात सहभागी होत आहे आणि ह्या सर्वेक्षणाबद्दलची माहिती आमच्या बोलीभाषेत व्यवस्थितपणे समजावून सांगितली आहे. ती माहिती पूर्णपणे गोपनीय राहिल. मी कधिही ह्या सर्वेक्षणातून काहीही कारण न देता बाहेर पडू शकते.

सहभागी झालेल्या व्यक्तीचे नाव | सहभागी झालेल्या व्यक्तिची सही

मुलाखत घेणाऱ्याचे नाव | मुलाखत घेणाऱ्याची सही
