# Supplementary material for: Undernutrition among tribal children in Palghar district, Maharashtra, India
Source: PLoS One. 2019 Feb 27;14(2):e0212560. doi: 10.1371/journal.pone.0212560 (PMC6392283; doi:10.1371/journal.pone.0212560)
Supplement: S2 Tool — (DOCX) [file pone.0212560.s003.docx]

| DATE | PHC | AWC | SUB-CENTRE | VILLAGE | Pada |  |
| --- | --- | --- | --- | --- | --- | --- |
|  |  |  |  |  |  |  |

**ANTHROPOMETRIC DATA**

| Sr.No. | 1.NAME OF CHILD | 2.AGE | 3.SEX | 4.Delivery after___ months of pregnancy | 5.BIRTH WT. | 6.HT. | 7.Wt. | 8.Birth Order |
| --- | --- | --- | --- | --- | --- | --- | --- | --- |
|  |  |  |  |  |  |  |  |  |
|  |  |  |  |  |  |  |  |  |

**SOCIOECONOMIC DATA**

| **9.** | Tribe 1.Mahadev Koli 2.Katkari 3. Warli 4.Others | | | |
| --- | --- | --- | --- | --- |
| 11. | Type of house | 1.Kuccha | 2.Semi Pucca | 3.Pucca |
| 12. | Lighting at home 1.Electricity 2. Kerosene lamp 3.Others _____________ | | | |
| 13. | Defecation practice | 1.Home toilet | 2.Community | 3.Fields |
| 14. | Source of drinking water | 1.Piped 2.Well(p) 3.Hand pump  4.Surface water 5.Others | | |
| 15. | Treatment of drinking water | 1.Filter A. cloth B. Candle  2.Boiling 3.Alum 4.Others 5.None | | |
| 16. | Use of iodised salt | 1.Yes 2.No | | |
| 17. | Fuel for cooking | 1.Wood 2.Kerosene stove 3.LPG  4.Dung cakes 5.Others | | |
| 18. | Place for cooking | 1. Kitchen 2.Courtyard 3.Unvented living area | | |
| 20. | Hand washing practices mother | 1.Soap 2.Ash 3.Only water 4.Other | | |
| 21. | Hand washing practices of child 1.Soap 2.Ash 3.Only water 4.Other | | | |
| 22. | Migration for work? Months? 1.Yes 2.No | | | |
| 23. | Did you migrate last year? 1.Yes 2.No | | | |
| 24. | How many times did you migrate in the last 5 years? | | | |
| 25. | Does the entire family migrate? 1.Yes 2.No | | | |
| 26. | Do you receive any benefits from Govt. schemes when you migrate? 1.Yes 2.No | | | |
| 27. | How do you immunize children when you migrate? | | | |
| 28. | Has your child ever been classified as malnourished?When? | | | |
|  |  | | | |

**DEMOGRAPHIC DATA**

| 29. | No. of household members | |  |  |  |  |
| --- | --- | --- | --- | --- | --- | --- |
| No. | Name | Age(completed  Years) | Sex  1.M 2.F | Relation to head of family | Education (No. of yrs of schooling) | Occupation |
|  |  |  |  |  |  |  |
|  |  |  |  |  |  |  |
|  |  |  |  |  |  |  |
|  |  |  |  |  |  |  |
|  |  |  |  |  |  |  |
|  |  |  |  |  |  |  |
|  |  |  |  |  |  |  |
|  |  |  |  |  |  |  |
|  |  |  |  |  |  |  |

1. Self-employed in agriculture 2.Self-employed in non-agriculture

3. Casual labourer in agriculture 4.Casual labourer in non-agriculture

5. Salaried/Wages (regular) 6.Unemployed

**CONSUMPTION EXPENDITURE**

| 30. | Food in 30 days | Clothes, bedding footwear in 365 days | Education in 365 days | Medicines in 365 days | Miscellaneous | Total |
| --- | --- | --- | --- | --- | --- | --- |
|  |  |  |  |  |  |  |

**BREAST-FEEDING PRACTICE**

|  |  |  | Child 1 | Child 2 |
| --- | --- | --- | --- | --- |
| 31. | Interval from previous birth |  |  |  |
| 32. | When did you start breast-  feeding after birth? | 1.Immediately  2.Within ½ hr  3.Within 24 hrs  4.More than 24 hrs |  |  |
| 33. | If not what was given |  |  |  |
| 34. | Was colostrum given to child? | 1.Yes 2.No |  |  |
| 35. | Was exclusive breast feeding done for 6months? | 1.Yes 2.No |  |  |
| 36. | If not reason  What was given? |  |  |  |
| 37. | Frequency of feeding in a day. |  |  |  |
| 38. | How long did you continue? |  |  |  |
| 39. | Did you get information about breast feeding?  By whom? | 1.AWW  2.ASHA  3.DAI | 4.ANM  5.DOCTOR  6.OTHERS |  |

**WEANING**

| 40. | Age of weaning |  |  |  |
| --- | --- | --- | --- | --- |
| 41. | Reason if late |  | | |
| 42. | Type of food given | 1. Liquid A. Rice water B. Dal water C. Milk D. Others |  |  |
|  |  | 2. Semi-Solid A. Kanji B. Khimti |  |  |
|  |  | 3. Solids A .Rice dal B. Roti-vegetables C. Eggs D. Others |  |  |

**IMMUNIZATION DETAILS**

|  |  | Child 1 | Child 2 |
| --- | --- | --- | --- |
| 43. | Did the child receive vaccine? 1.Y 2.N |  |  |
| 44. | BCG & Polio at birth  OPV1 +DPT1 (6Wks)Pentavalent  OPV2+DPT2 (10Wks)  OPV3+DPT3 (14Wks)  Measles 1 (9-12m)  2 (16-24m) |  |  |
|  |  |  |  |
|  |  |  |  |
|  |  |  |  |
| 45. | Vitamin A (9m onwards) 1.Y 2.N |  |  |
| 46. | Deworming 1.Y 2.N |  |  |
| 47. | Where is immunization done? |  |  |

**ILLNESS**

| 48. | Any illness in last 1 month |  |  |  |
| --- | --- | --- | --- | --- |
| 49. | Can you name it? 1.Diarrhea  2.Fever 3.Cough & cold | |  |  |
| 50. | Where did you take the child 1.Public facility 2.Private doctor 3.Traditional healer | |  |  |
| 51. | How soon after symptoms |  |  |  |
|  | Was the child referred to any centre? |  |  |  |
| 52. | Was the child hospitalised in past 12 m? |  |  |  |
| 53. | Any chronic illness? |  |  |  |

**UTILISATION OF SOCIAL SECURITY SCHEMES**

| 54. | Is AWC present in the village? 1.Yes 2.No |  |  |
| --- | --- | --- | --- |
| 55. | Distance of AWC from home |  |  |
| 56. | Is the child enrolled in AWC 1.Yes 2.No |  |  |
| 57. | What food supplement is provided  1.Dry snack /m  2.THR /m  3Hot Cooked Meal /m  4.Fruit /m  5.Eggs /m |  |  |
| 58. | No. of times health check-up done in AWC? |  |  |
|  | No. of times the child was weighed in AWC. |  |  |
| 59. | No. of days /m the child go to AWC |  |  |
| 60. | Do you get ration from PDS? |  |  |
|  | Do you have Antyodaya card |  |  |
| 61. | Did you get benefits from JSY? |  |  |
|  | Matrutva Anudaan Yojana |  |  |
|  | MGNREGS, EGS? |  |  |

**MOTHER RELATED DETAILS**

| 62.  Age | 63. Age at marriage | 64.Age at 1^st^ pregnancy | 65.Total no. of pregnancies | 66.no.of live births | 67.ANC visits | 68.IFA Tablets taken | 69.TT injections | 70.Place of delivery |
| --- | --- | --- | --- | --- | --- | --- | --- | --- |
|  |  |  |  |  |  |  |  |  |
|  |  |  |  |  |  |  |  |  |

| 71. | Special supplements provided | 1.Yes 2.No |  |
| --- | --- | --- | --- |
| 72. | PNC check-up | 1.Yes 2.No |  |
| 73. | Advice given during pregnancy by Asha | 1.Yes 2.No |  |
| 74. | Spacing between children   1. 1^st^ and 2^nd^ child 2. 2^nd^ and 3^rd^ child 3. 3^rd^ and 4^th^ child |  |  |
| 75. | Current family planning method 1.OC Pills 2.Copper T 3.Condoms 4.TL | | |
| 76. | Do you have any habits? 1.Tobacco _______________ 2.Alcohol 3.others | | |

**CHILD DIET**

**77.** Is the child given same meal as family? 1. Yes 2.No

| Meals in last 24hr | Name of food item eaten | Ingredients | Total amt. consumed in grams | Amount consumed in calories |
| --- | --- | --- | --- | --- |
| Breakfast | 1. |  |  |  |
|  |  |  |  |  |
|  | 2. |  |  |  |
|  |  |  |  |  |
|  | 3. |  |  |  |
| Lunch | 1. |  |  |  |
|  |  |  |  |  |
|  | 2. |  |  |  |
|  |  |  |  |  |
|  | 3. |  |  |  |
| Snacks | 1. |  |  |  |
|  |  |  |  |  |
|  | 2. |  |  |  |
|  |  |  |  |  |
|  | 3. |  |  |  |
| Dinner | 1. |  |  |  |
|  |  |  |  |  |
|  | 2. |  |  |  |
|  |  |  |  |  |
|  | 3. |  |  |  |
| others | 1.  2. |  |  |  |
